# SPINT2 Is Associated with Dual-Phenotype Hepatocellular Carcinoma and Aggressive Tumor Features

**DOI:** 10.3390/cancers18193102

**Published:** 2026-09-24

**Authors:** Jie Zhang, Minjun Li, Taixin Yang, Ning Ma, Yaohong Liu, Chunye Fang, Chenglei Yang, Bangde Xiang

**Affiliations:** 1 Department of Hepatobiliary Surgery, Guangxi Medical University Cancer Hospital, Nanning 530021, China; zhangjie1@gxmu.edu.cn (J.Z.);; 2 Guangxi Key Laboratory of Early Prevention and Treatment of Regional High Frequency Tumors, Nanning 530021, China; 3 Division of Health Science, Graduate School of Health Science, Suzuka University of Medical Science, Suzuka 510-0293, Japan; maning@suzuka-u.ac.jp; 4 School of Oncology, Guangxi Medical University, Nanning 530021, China

**Keywords:** dual-phenotype hepatocellular carcinoma, SPINT2, stemness

## Abstract

**Background**: Hepatocellular carcinoma (HCC) heterogeneity, particularly the presence of tumor cells co-expressing hepatocytic and cholangiocytic markers, contributes to therapeutic resistance and poor prognosis, yet its molecular correlates remain incompletely understood. This study aimed to characterize dual-phenotype HCC (DPHCC) and investigate the association and functional relevance of SPINT2 in tumor progression. **Methods**: We integrated multiplex immunofluorescence, CyTOF mass cytometry, single-cell RNA sequencing, and functional assays across clinical cohorts and cell lines. **Results**: Multiplex staining identified DPHCC in 22.89% of patients, correlating significantly with younger age, elevated AFP, and inferior recurrence-free and overall survival compared to non-DPHCC cases. Parallel scRNA-seq and CyTOF analyses showed that DPHCC-associated cells were enriched for stemness- and EMT-related features, with SPINT2 emerging as a top candidate gene in the transcriptomic analysis. Immunohistochemistry showed higher SPINT2 expression in DPHCC tissues, and high SPINT2 expression was associated with shorter overall survival in univariate Kaplan–Meier analysis and frequently co-localized with CK19. Functionally, SPINT2 modulation produced context-dependent changes in invasion, proliferation-related CCK-8 readouts, wound closure, sphere formation, and xenograft tumor growth in MHCC-97H and MHCC-LM3 cells. Collectively, these findings characterize DPHCC as a clinically relevant dual-lineage phenotypic state associated with aggressive tumor features and identify SPINT2 as a candidate molecule associated with this phenotype. **Conclusions**: SPINT2 is associated with the DPHCC-related state and aggressive tumor-cell phenotypes, but its mechanistic and clinical significance requires further validation.

## Simple Summary

Liver cancer displays substantial tumor-cell heterogeneity, including tumor cells that simultaneously express hepatocytic and cholangiocytic markers. This study characterizes this dual-lineage phenotype in hepatocellular carcinoma and investigates its association with SPINT2. By integrating patient tissue profiling, single-cell analyses, and functional experiments, we show that this phenotype is associated with adverse clinical outcomes and aggressive tumor features. SPINT2 is further identified as a candidate molecule associated with these malignant phenotypes. These findings provide insight into HCC cellular plasticity and the biological heterogeneity of dual-lineage tumor states.

## 1. Introduction

Hepatocellular carcinoma (HCC) remains a leading cause of cancer-related mortality worldwide, driven largely by its profound intratumoral heterogeneity, which underpins therapeutic resistance and poor clinical outcomes [1]. Among the heterogeneous phenotypic states observed in HCC, CK19-positive or progenitor-like tumors may exhibit biliary/progenitor-marker expression while retaining an otherwise hepatocellular phenotype [2]. Related studies have further characterized the histological and cholangiocellular heterogeneity of primary liver tumors and HCC [3,4,5]. HCC stemness and stem-like cellular hierarchies have also been increasingly characterized using transcriptomic and single-cell approaches [6,7,8]. SPINT2 has been implicated in protease-regulatory biology and has shown context-dependent effects across different disease and cancer models [9,10]. Molecular-subtype analyses and recent clinical studies have further highlighted HCC heterogeneity and the aggressive clinical behavior of DPHCC [11,12,13]. In contrast, dual-phenotype HCC (DPHCC) is characterized by same-cell co-expression of hepatocytic and cholangiocytic markers rather than CK19 positivity alone [14,15,16]. Previous work has explicitly distinguished DPHCC from CK19-positive HCC and from tumors showing spatially separate hepatocellular and cholangiocarcinoma components or non-overlapping hepatocytic and cholangiocytic marker expression [14,16]. Accordingly, DPHCC is considered here as a dual-lineage immunophenotypic state within HCC and should not be regarded as synonymous with either CK19-positive/progenitor-like HCC or combined hepatocellular–cholangiocarcinoma (cHCC-CCA). The presence of such dual-lineage tumor cells may reflect increased phenotypic plasticity and progenitor-like features and may contribute to aggressive tumor behavior and adverse clinical outcomes.

Despite the recognized clinical aggressiveness of DPHCC, the precise prevalence, molecular correlates and candidate regulators, and prognostic significance of DPHCC cells within typical HCC have remained poorly defined [16]. While single-cell technologies have begun to unravel HCC heterogeneity, a systematic multimodal characterization of DPHCC—from single-cell transcriptomics and proteomics to functional validation—has been lacking. Furthermore, the specific genes and pathways that are associated with this dual-phenotype state and its associated stemness [8] are largely unknown. The serine protease inhibitor Kunitz type 2 (SPINT2) gene, a known inhibitor of matriptase and other proteases [9], presents a complex and context-dependent role in cancer, acting as a tumor suppressor in some settings [10] while potentially promoting pathological processes in others. Its role in the context of DPHCC and HCC stemness has not been explored, representing a critical knowledge gap.

To address this gap, the present study employs a multimodal profiling approach. We combine advanced multiplex immunofluorescence and traditional immunohistochemistry to define and quantify DPHCC cells in a clinical HCC cohort. This is complemented by high-dimensional single-cell proteomics using CyTOF mass cytometry and single-cell RNA sequencing (scRNA-seq) to delineate the transcriptomic and proteomic landscape of DPHCC cells, with particular emphasis on stemness and developmental pathways. Furthermore, the functional effects of the candidate gene SPINT2 were evaluated through a series of in vitro assays (including invasion, proliferation, wound closure, and sphere formation) and in vivo xenograft models. The specific purpose of this study is threefold: (1) to determine the prevalence, clinical features, and prognostic impact of DPHCC in HCC patient cohort; (2) to identify key differentially expressed genes, particularly SPINT2, and the signaling pathways that characterize DPHCC; and (3) to evaluate the functional effects of SPINT2 modulation on aggressive tumor-cell phenotypes, including sphere-forming capacity and to assess its potential biological relevance to the DPHCC-associated state.

## 2. Materials and Methods

### 2.1. Materials

This study was approved by the Scientific Ethics Committee of the Affiliated Tumor Hospital of Guangxi Medical University (Ethic Committee Name: Scientific Ethics Committee of the Affiliated Tumor Hospital of Guangxi Medical University; Approval Code: KY2025575; Approval Date: 23 July 2025). This approval covered the retrospective analysis of archived clinical specimens and associated clinical data collected from patients who underwent liver resection between 2018 and 2019. Human HCC cell lines MHCC-97H (SCSP-5092) and MHCC-LM3 (SCSP-5093) were purchased from the National Cell Resource Center, Chinese Academy of Sciences. Four-week-old male BALB/c nude mice were purchased from the Guangxi Medical University Animal Center.

### 2.2. Follow-Up Protocol

A total of 83 patients with HCC who underwent liver resection at the Affiliated Tumor Hospital of Guangxi Medical University between May 2018 and July 2019 were retrospectively enrolled. Inclusion criteria were histopathologically confirmed HCC after liver resection, availability of adequate archived tumor tissue for pathological evaluation, and complete clinicopathological and follow-up information. Exclusion criteria were combined hepatocellular–cholangiocarcinoma or other concurrent malignancies, inadequate tumor tissue for pathological evaluation, or incomplete clinical or follow-up data. Tumor stage was recorded according to the Barcelona Clinic Liver Cancer (BCLC) classification. Patients who had received preoperative antitumor treatment were excluded. Postoperative treatment, when clinically indicated, was administered according to routine clinical practice and individual disease status rather than according to a study-mandated protocol. Patients were followed up via outpatient visits or telephone interviews. Assessments included tumor recurrence, recurrence time, and overall survival. Follow-up visits were scheduled monthly for the first 3 months, every 3 months thereafter for 2 years, and every 6 months after that. Recurrence-free survival (RFS) was defined as the time from surgery to recurrence, and overall survival (OS) as the time from surgery to death or the last follow-up (31 March 2025).

### 2.3. Multiplex Immunofluorescence (mIF) Staining

The primary antibodies included hepatocyte markers Hep Par 1, Glypican-3, Arginase-1 and cholangiocyte markers CK7 and CK19. Staining was performed on formalin-fixed, paraffin-embedded (FFPE) tumor tissue sections. Tumor cell phenotype was determined at the single-cell level. Image analysis was performed using Phenochat 1.0 (Akoya Biosciences, Inc., Billerica, MA, USA). Tumor regions were identified on the corresponding H&E-stained sections based on tumor morphology, and normal biliary/ductular structures and non-tumor regions were excluded before quantitative analysis. Fluorescence intensity was quantified at the single-cell level, and marker-specific positivity thresholds were applied within the software-assisted quantitative workflow. For each sample, ten random regions (approximately 5000 cells per region) were selected. The positivity rate for each antibody was calculated. At the single-cell level, a DPHCC cell was operationally defined as a tumor cell simultaneously positive for at least one hepatocytic marker (Hep Par 1, GPC-3, or Arg-1) and at least one cholangiocytic marker (CK7 or CK19). The DPHCC-cell fraction for each tumor was calculated as the number of dual-positive tumor cells divided by the total number of evaluated tumor cells. For the primary analysis, tumors with a DPHCC-cell fraction >5% were classified as DPHCC-positive. Because no universally accepted quantitative threshold for DPHCC has been established, the 5% threshold was treated as a study-specific operational cutoff rather than as a universal pathological diagnostic criterion. A 5% cutoff has previously been used to define CK19-positive HCC based on CK19 positivity alone. In the present study, however, the 5% threshold was applied to tumor cells showing same-cell co-expression of hepatocytic and cholangiocytic markers, representing a more stringent phenotypic requirement than CK19 positivity alone. Previous DPHCC studies used a higher numerical threshold (>15%) for dual-positive cells [14,16]. To assess the dependence of case classification on the selected threshold, sensitivity analyses were additionally performed using alternative cutoffs of >1%, >3%, >10%, and >15%.

### 2.4. Mass Cytometry (CyTOF) Analysis

Single-cell suspensions were prepared from fresh tumor samples (*n* = 16) using collagenase digestion and red blood cell lysis. These 16 cases represented a fresh-tissue subset of the 83-patient clinical cohort. Each patient-derived tumor sample was treated as one independent biological replicate. Cells were stained with a panel of lanthanide-conjugated antibodies targeting surface and intracellular markers. The analyzed panel included the lineage markers Hep Par1 and CK19 together with CSC- and tumor-associated markers including CD326 (EpCAM), p53, ALDH, CD325, CD34, AFP, OV6, CD133, LGR5, CD90, p21, CD13, DNMT3B, CD24, MUC1, NANOG, CD54, CD274, and c-Myc. Nucleic acid was labeled with Cell-ID™ Intercalator-Ir. For the CyTOF-level DPHCC analysis, gating thresholds were applied in Cytobank, and cells simultaneously positive for Hep Par1 and CK19 were operationally classified as dual-positive DPHCC cells (Section 3). Data were acquired on a Helios™ 2 system at <500 events/second and normalized using the standard instrument-associated normalization workflow before downstream analysis. No separate batch-correction algorithm was applied after normalization. The resulting data were processed on the Cytobank platform. FlowSOM clustering based on the CSC- and tumor-associated marker set described above yielded 17 phenotypic clusters, which were visualized by UMAP for downstream comparison.

### 2.5. Single-Cell RNA Sequencing (scRNA-Seq)

Fresh tumor tissues from 8 patients (2019–2020) were enzymatically dissociated to generate single-cell suspensions. These eight scRNA-seq cases constituted an independent cohort and did not overlap with either the 83-patient clinical cohort or the 16-patient CyTOF subgroup. Library construction and sequencing were performed by a commercial service. Raw data were processed for quality control, alignment, and expression matrix generation. Cells were retained if they had >500 and <2500 detected genes, <15,000 total RNA counts, <25% mitochondrial transcripts, and <1% hemoglobin-gene content. Potential doublets were identified at the individual-sample level using Scrublet and excluded before downstream analyses. To assess the sensitivity of dual-lineage classification to low-level ambient transcripts, we additionally repeated the analysis using a more stringent within-cell expression requirement for both hepatocytic and cholangiocytic marker sets. The Seurat workflow was used for normalization, batch effect correction (FastMNN), and cell clustering (UMAP). Cell types were annotated based on canonical markers. Malignant cells were identified via copy number variation (CNV) analysis. A broader DPHCC-associated transcriptional state was identified based on hepatocytic and cholangiocytic lineage-marker expression. To assess the presence of bona fide dual-lineage cells within this state, we additionally performed a stricter marker-level sensitivity analysis requiring detectable expression of both hepatocytic and cholangiocytic marker sets within the same malignant cell. Pseudotime analysis (Monocle) and RNA velocity (Velocyto) were performed to infer developmental trajectories. To account for the non-independence of cells from the same patient, differential-expression analysis was additionally performed using a donor-aware pseudobulk approach. Raw counts were aggregated by patient and cell state, and differential expression was tested using edgeR with patient identity included in the design matrix. Because marker availability differed across modalities, mIF, CyTOF, and scRNA-seq used modality-specific operational definitions; these analyses were therefore interpreted as complementary rather than as identical phenotype classifications.

### 2.6. Cell Lines and Culture

Human HCC cell lines MHCC-97H and MHCC-LM3 were purchased from the Cell Bank of the Chinese Academy of Sciences. Cells were cultured in DMEM medium supplemented with 10% fetal bovine serum (FBS) and 1% penicillin/streptomycin at 37 °C in a 5% CO_2_ incubator. For gene function studies, MHCC-97H and MHCC-LM3 cells were stably transduced with lentiviral vectors for SPINT2 overexpression (OE) or knockdown (SH), along with their respective negative controls (NC). For SPINT2 knockdown, three lentiviral shRNA constructs were generated using the GV493 vector (hU6-MCS-CBh-gcGFP-IRES-puromycin; GeneChem, Shanghai, China): SPINT2-RNAi (123258-1), 5′-CAGCTGGTGAAGAACACATAT-3′; SPINT2-RNAi (123259-1), 5′-CTCCAGCGATATGTTCAACTA-3′; and SPINT2-RNAi (123260-1), 5′-CCAGCAGGAATGCAGCGGATT-3′. The non-targeting control sequence was 5′-TTCTCCGAACGTGTCACGT-3′ (CON313). Stable transfectants were selected using puromycin. Of the three SPINT2-targeting constructs, one selected shRNA construct was used for the downstream functional experiments.

### 2.7. In Vivo Xenograft Assay

All animal experiments were approved by the Institutional Animal Care and Use Committee. Four- to five-week-old male BALB/c nude mice were housed under specific-pathogen-free (SPF) conditions. A total of 1 × 10^7^ cells (100 μL) from each stable cell line (OE, OE-NC, SH, SH-NC; *n* = 4/group) were subcutaneously injected into the right shoulder of the mice. Tumor growth was monitored every 3–4 days, and tumor volume was calculated as (length × width^2^)/2. Mice were sacrificed on day 15, and tumors were excised and weighed for final analysis.

### 2.8. Histological and Protein Analysis

Immunohistochemistry (IHC): FFPE sections were deparaffinized, rehydrated, and subjected to antigen retrieval. After blocking, sections were incubated with primary antibodies against SPINT2, CK19, and other targets overnight at 4 °C, followed by HRP-conjugated secondary antibody incubation. DAB was used for visualization, and slides were counterstained with hematoxylin. Protein expression was scored by two independent pathologists; >25% positive tumor cells was defined as high expression.

### 2.9. In Vitro Functional Assays

Cell Proliferation (CCK-8): Cells were seeded in 96-well plates (4 × 10^3^ cells/well). At indicated time points (0, 24, 48, 72, and 96 h), CCK-8 reagent was added, and absorbance at 450 nm was measured.

Colony Formation: Cells (1 × 10^3^ cells/well) were seeded in 6-well plates and cultured for 16 days. Colonies were fixed, stained with crystal violet, and counted using ImageJ version 1.8.0.345.

Transwell Invasion Assay: Transwell inserts were coated with Matrigel. Serum-starved cells (1 × 10^5^ cells/well) were seeded in the upper chamber, and the lower chamber contained 10% FBS as a chemoattractant. After 24 h, invaded cells were fixed, stained, and counted.

Wound Healing Assay: Confluent cell monolayers were scratched with a 200 μL pipette tip. Cells were washed and cultured in serum-free medium. Wound closure was imaged at 0, 24, and 48 h, and the wound-closure rate was calculated using ImageJ version 1.8.0.345.

Tumor Sphere Formation: Cells were cultured in ultra-low attachment plates in serum-free medium containing growth factors. After 6–7 days, tumor spheres (>50 μm in diameter) were counted and photographed.

### 2.10. Statistical Analysis

Statistical analyses were performed using SPSS 23.0 and R 4.3.1. Categorical variables were compared using the chi-square test or Fisher’s exact test. Continuous variables were analyzed by Student’s *t*-test or ANOVA. Survival curves (RFS and OS) were estimated using the Kaplan–Meier method, and differences were assessed by the log-rank test. Univariate and multivariate Cox proportional hazards regression models were used to evaluate prognostic associations. Variables with *p* < 0.05 in univariate Cox regression analyses were entered into the corresponding multivariable Cox models. Model complexity was assessed using events per variable (EPV), multicollinearity among covariates was assessed using variance inflation factors (VIFs), and the proportional hazards assumption was evaluated using Schoenfeld residuals. Unless otherwise stated, in vitro experiments were performed in three independent biological replicates, and data are presented as mean ± SD. Two-group comparisons were performed using two-tailed unpaired Student’s *t*-tests. CCK-8 time-course data were analyzed using two-way ANOVA followed by Šídák’s multiple-comparisons test. A two-tailed *p* < 0.05 was considered statistically significant.

## 3. Results

### 3.1. Phenotypic Characteristics of Five-Antibody Fluorescent Staining and HE Staining

Hep par 1 (Hep1), Glypican-3 (GPC-3), Arginase-1 (Arg-1), CK7, and CK19 all showed positive expression in 83 tumor tissue samples, with positive expression rates of 88.0% (73/83), 88.0% (73/83), 86.7% (72/83), 8.4% (7/83), and 21.7% (18/83), respectively. Based on their fluorescence intensity, the staining of the five antibodies can simulate immunohistochemical staining to observe the positivity of the five antibody stainings on the same slide (Figure 1A). Through single-cell pathological scanning, the positive expression of multiple antibodies on the same tumor cell can be localized. The co-expression positive rates of Arg-1 + CK7, Arg-1 + CK19, Gly-3 + CK7, Gly-3 + CK19, Hep1 + CK7, and Hep1 + CK19 were 1.2% (1/83), 3.6% (3/83), 4.8% (4/83), 19.3% (16/83), 2.4% (2/83), and 7.2% (6/83), respectively, with an overall positive rate of 22.89% (19/83). Using the primary operational cutoff of >5%, 19 of 83 tumors (22.9%) were classified as DPHCC-positive (Figure 1B). Sensitivity analyses showed that 36/83 (43.4%), 22/83 (26.5%), 19/83 (22.9%), 10/83 (12.0%), and 6/83 (7.2%) tumors were classified as DPHCC-positive at cutoffs of >1%, >3%, >5%, >10%, and >15%, respectively. After phenotypic statistics following multiplex immunofluorescence staining, the expression levels of hepatocyte markers Arg-1 and Hep1 in DPHCC tumor cells were lower than those in non-DPHCC (*p* < 0.05), while the expression levels of cholangiocyte markers CK7 and CK19 were higher than those in non-DPHCC (*p* < 0.05).

### 3.2. Clinicopathological and Prognostic Characteristics of DPHCC Patients

Differences in clinicopathological baseline data between DPHCC and non-DPHCC included indicators such as gender, age (>60 years), BMI, diabetes, tumor capsule, multiple nodules, maximum tumor diameter, BCLC stage, liver cirrhosis, MVI, Edmonson grade, HBsAg qualitative status, PT time (>13.0 s), AFP (>400 ng/mL), and albumin (<35 g/L). Results showed that only 2 cases (10.5%) in DPHCC patients were older than 60 years, a proportion lower than the 24 cases (37.5%) in non-DPHCC patients, with a *p* value of 0.026. There were 12 cases (63.2%) of patients with AFP > 400 ng/mL, a proportion higher than the 20 cases (31.3%) in non-DPHCC patients. DPHCC patients were younger and had higher serum Alpha Fetoprotein (AFP) levels (Table 1).

Kaplan–Meier analysis showed that patients with DPHCC had significantly poorer RFS and OS than patients with non-DPHCC tumors. The 1-, 3-, and 5-year RFS estimates were 47.4%, 21.1%, and 21.1%, respectively, in the DPHCC group and 67.2%, 42.2%, and 35.3%, respectively, in the non-DPHCC group. The corresponding 1-, 3-, and 5-year OS estimates were 68.4%, 31.6%, and 31.6% in the DPHCC group and 89.1%, 67.2%, and 57.4% in the non-DPHCC group. The between-group differences were significant for both RFS (log-rank *p* = 0.026) and OS (log-rank *p* = 0.005) (Figure 1C).

### 3.3. Risk Factors Related to Prognosis in the Sample Cohort

This study further analyzed prognostic risk factors for 83 HCC patients meeting the inclusion criteria using Cox regression, including risk factors affecting HCC patient recurrence (Table 2) and risk factors affecting HCC patient overall survival (Table 3).

Univariate analysis results regarding factors affecting recurrence in 83 HCC patients showed that multiple nodules, Edmonson grade (Grade III-IV), neutrophil-to-lymphocyte ratio (NLR > 2.51), platelet-to-lymphocyte ratio (PLR > 180), and DPHCC phenotype were all significantly associated with HCC recurrence. Further multivariable Cox regression analysis showed that multiple nodules, PLR > 180, and the DPHCC phenotype remained associated with recurrence after adjustment for the variables included in the model. After correcting for other potential confounding factors, these three variables remained significant, suggesting they may serve as important prognostic indicators in clinical practice.

Univariate analysis results of risk factors for overall survival in 83 HCC patients showed that incomplete capsule, Edmonson grade (Grade III-IV), PLR > 180, AFP > 400 ng/mL, and DPHCC phenotype were all significantly associated with lower overall survival in HCC patients (*p* < 0.05). In further multivariate analysis, Edmonson grade (Grade III-IV), PLR > 180, and DPHCC phenotype remained associated with OS after adjustment for the variables included in the model.

In summary, the multivariable analyses showed that the DPHCC phenotype was associated with adverse RFS and OS after covariate adjustment. During follow-up, 57 RFS events and 41 deaths were observed. Five variables were entered into each multivariable Cox model, corresponding to EPV values of 11.4 for RFS and 8.2 for OS. Schoenfeld residual-based assessment did not indicate significant violations of the proportional hazards assumption, and no substantial multicollinearity was observed among the covariates (all VIFs < 1.4). Given the cohort size, these multivariable results are interpreted as adjusted associations rather than definitive prognostic validation.

### 3.4. Mass Cytometry Expression Profile of DPHCC Tumor Cells

Among the 83-patient clinical cohort, fresh tumor tissues from 16 patients were available for mass cytometry analysis. Gate operations were performed according to thresholds calibrated by Cytobank for cell extraction and grouping. The expression status of diagnostic markers Hep par 1 and CK19 in the included samples is shown in Figure 2A. Single-cell mass cytometry analysis classified all tumor cells into 17 clusters. Among them, DPHCC tumor cells were mainly enriched in clusters 13 and 15 (Figure 2B,C).

Comparing all DPHCC tumor cells and non-DPHCC tumor cells, among the 29 detected markers, 22 markers were significantly highly expressed. Except for 2 diagnostic markers (Hep par 1, CK19), the remaining 19 CSC markers were significantly highly expressed, as detailed in Figure 2D.

### 3.5. Velocyto Velocity Analysis and Pseudotime Analysis

To determine whether the broader DPHCC-associated transcriptional state contained cells with a strict dual-lineage profile rather than reflecting cholangiocytic/progenitor-marker expression alone, we performed an additional marker-level sensitivity analysis. A total of 614 of 8873 malignant cells (6.9%) showed detectable expression of both hepatocytic and cholangiocytic marker sets, accounting for 56.4% (614/1089) of the broader DPHCC-associated state; all strict dual-lineage cells were contained within this broader state. When a more stringent within-cell expression requirement was applied to reduce sensitivity to low-level ambient transcripts, 182 of 8873 malignant cells (2.1%) remained positive for both lineage-marker sets.

The broader DPHCC-associated transcriptional state was mainly distributed in Cluster 0, Cluster 3, Cluster 4, Cluster 6, and Cluster 7 subpopulations, generally divided into two regions (Figure 2E). RNA velocity analysis suggested distinct patterns of inferred transcriptional direction across the DPHCC-enriched clusters, with Clusters 0/4 and Clusters 3/6/7 showing different vector orientations (Figure 2F). Monocle pseudotime analysis placed DPHCC-associated cells in different regions of the inferred trajectory (Figure 2G). These analyses represent computational inferences of transcriptional dynamics and do not establish definitive developmental transitions or lineage directionality.

### 3.6. Differential Gene Analysis Between DPHCC and Non-DPHCC Tumor Cells

Single-cell transcriptomic analysis was performed to screen DPHCC and non-DPHCC cells based on the differential expression of KRT7, KRT19, CPS1, and GPC3 (Figure 2H). The FindAllMarkers function was used to compare gene expression differences between DPHCC and non-DPHCC group cells. Screening was performed based on adjusted *p* value < 0.01 and log2FC value 0.05, obtaining a total of 518 differentially expressed genes. The TOP4 genes were selected to draw a heatmap, as shown in Figure 3A. The TOP10 genes were SPINT2, KRT19, EpCam, KRT7, ALB, GC, APOC1, FGB, UQCRQ, and TTR. Because KRT7 and KRT19 contributed to the operational definition of the DPHCC-associated state, their differential expression was considered expected and was not interpreted as independent validation of the transcriptional signature. Among them, the expression level differences of CSC markers between DPHCC and non-DPHCC cells were compared. The results showed that EpCam, CDH2, AFP, TP53, LGR5, THY1, ANPEP, DNMT3B, CD24, MUC1, and ICAM1 were significantly highly expressed in DPHCC cells, as shown in Figure 2I. After quality control and doublet removal, 8873 malignant cells from eight patients were retained for analysis, with patient-specific cell numbers and contributions to the DPHCC-associated and non-DPHCC states summarized in Supplementary Table S1. Substantial inter-patient heterogeneity was observed. Because cells from the same patient are not independent biological replicates, we additionally performed a donor-aware pseudobulk analysis using patient identity as the biological replicate. SPINT2 retained a positive association with the DPHCC-associated state after accounting for patient identity (log2FC = 0.60, nominal *p* = 0.030), although the association did not remain significant after transcriptome-wide FDR correction (FDR = 0.630). A sensitivity analysis restricted to patients with at least 20 cells in both states yielded a similar effect size (log2FC = 0.62, *p* = 0.0076), but the association likewise did not remain significant after FDR correction (FDR = 0.561). Accordingly, the original cell-level differential-expression analysis was treated as exploratory, while the donor-aware pseudobulk analysis provided the patient-level statistical assessment (Supplementary Table S2).

### 3.7. GSEA Pathway Enrichment Analysis

To further analyze the DPHCC phenotype and its occurrence and development, as well as pathways and biological mechanisms related to CSC marker expression, this study further adopted the GSEA (Gene Set Enrichment Analysis) method for exploratory pathway-level characterization. Through gene ranking, cumulative statistic calculation, and permutation tests, the synergistic enrichment degree of DPHCC and non-DPHCC differential gene sets in the expression profile was evaluated.

GSEA enriched a total of 909 pathways. Among the top enriched pathways, several stemness-, epithelial-development-, motility-, and EMT-related gene sets were enriched in the DPHCC-associated state, including Desert Stem Cell HCC Subclass Up, GOBP Epithelium Development, GOBP Cell Motility, Onder CDH1 Targets 2, and Hollern EMT Breast Tumor (Figure 3B).

### 3.8. Histological Evaluation of SPINT2 Expression

To evaluate the expression difference of SPINT2 between DPHCC and non-DPHCC patients, this study employed immunohistochemical staining to analyze 83 tumor tissue samples. The samples included tumor tissues from DPHCC patients and non-DPHCC patients, and SPINT2 protein expression was detected in each. By scoring the staining results of tissue sections, the proportions of patients with high and low SPINT2 expression were determined.

Results showed that among 83 HCC tumor tissues, 12 patients exhibited high SPINT2 expression, while 71 patients exhibited low expression. Further analysis revealed that among these 12 high-expression patients, 9 were DPHCC patients and 3 were non-DPHCC patients. Specifically, the high expression rate of SPINT2 in DPHCC patients was 47.37%, whereas the high expression rate in non-DPHCC patients was only 4.7%. This result indicates that SPINT2 expression in DPHCC patients is significantly higher than in non-DPHCC patients (*p* < 0.001), as shown in Figure 3C.

### 3.9. Correlation Between SPINT2 Expression Level and Liver Cancer Prognosis

This study evaluated the impact of SPINT2 expression levels on HCC prognosis through Kaplan–Meier survival analysis. Survival curves revealed that although the Recurrence-Free Survival (RFS) between the SPINT2 high-expression group and the low-expression group did not reach the statistical significance threshold (log-rank *p* = 0.16), the RFS curve of the high-expression group showed a continuous downward trend (Figure 3D). Specifically, the postoperative 1-, 3-, and 5-year RFS rates for the low-expression subgroup were 64.8%, 39.4%, and 33.3%, respectively, while the RFS rates for the high-expression cohort at corresponding time points dropped to 41.7%, 25.0%, and 0%; however, the difference in RFS was not statistically significant.

In the Overall survival (OS) analysis, high SPINT2 expression was associated with shorter OS in univariate Kaplan–Meier analysis. The median OS in the high-expression group was 14.3 months shorter than that in the low-expression group (19.6 months vs 33.9 months), and the log-rank test showed that the difference between groups was statistically significant (*p* = 0.012). The corresponding survival estimates were consistent with this association: the postoperative 1-, 3-, and 5-year OS rates for the low-expression subgroup were 87.3%, 63.4%, and 54.6%, respectively, while the OS rates for the high-expression group at corresponding time points sharply decreased to 50.0%, 33.3%, and 16.7% (Figure 3D).

### 3.10. Co-Expression Status of SPINT2 and CK19

Results showed that the majority of SPINT2-positive tumor cells exhibited co-expression with CK19. Specifically, among the observed tumor cells, approximately 70% of SPINT2-positive cells simultaneously expressed CK19, a phenomenon verified across different tumor samples. This finding indicates frequent co-expression of SPINT2 and CK19 in HCC tumor cells (Figure 3E).

### 3.11. Effect of SPINT2 on the Invasion Ability of Liver Cancer Cells

Transwell invasion assay results showed that compared with the control group, the number of cells passing through the collagen film after 24 h was significantly increased in the 97H OE cell line and LM3 OE, indicating that SPINT2 overexpression significantly enhanced cell invasion ability, and the difference was statistically significant (*p* < 0.05). Compared with the control group, the number of cells passing through the collagen film after 24 h was significantly reduced in the 97H SH cell line and LM3 SH, showing that SPINT2 knockdown significantly inhibited cell invasion ability, and the difference was statistically significant (*p* < 0.05). Specifically, the cell count in the 97H SH group decreased by approximately 60% on average compared to the control group, while the cell count in the LM3 SH group decreased by approximately 55% on average (Figure 4A). These results further support the important role of SPINT2 in the cell invasion process; cells overexpressing SPINT2 exhibited stronger invasion ability, while knocking down SPINT2 significantly inhibited this ability.

### 3.12. Effect of SPINT2 on the Proliferation Ability of Liver Cancer Cells

The effect of SPINT2 on proliferative phenotypes was evaluated using colony-formation assays (Figure 4B) and CCK-8 assays (Figure 4D). In the colony-formation assay, colony numbers were significantly increased in the MHCC-97H OE group (*p* = 0.0154) and MHCC-LM3 OE group (*p* = 0.0384) compared with their corresponding controls. Conversely, colony numbers were significantly reduced in the MHCC-97H SH group (*p* = 0.0006) and MHCC-LM3 SH group (*p* = 0.0010) compared with their corresponding controls. These findings indicate that SPINT2 modulation altered colony-forming capacity in both HCC cell lines.

In the CCK8 assay, the 97H OE group showed higher readouts than the corresponding control at 48 h (adjusted *p* = 0.0053), 72 h (adjusted *p* < 0.0001), and 96 h (adjusted *p* < 0.0001), whereas the difference at 24 h was not significant (adjusted *p* = 0.0542). This result suggests that SPINT2 overexpression may promote the proliferation of 97H cells, indicating that SPINT2 has a potential positive regulatory function on cell proliferation. Meanwhile, the CCK-8 readout of the 97H SH group was higher than that of the corresponding control at 24 h (adjusted *p* = 0.0261), 72 h (adjusted *p* = 0.0122), and 96 h (adjusted *p* < 0.0001), whereas no significant difference was observed at 48 h (adjusted *p* = 0.2423).

For the LM3 cell line, the OE group showed higher CCK-8 readouts at 72 h (adjusted *p* = 0.0032) and 96 h (adjusted *p* < 0.0001), with no significant differences at 24 h (adjusted *p* = 0.9041) or 48 h (adjusted *p* = 0.9371); the SH group showed lower CCK-8 readouts at 48 h (adjusted *p* = 0.0002), 72 h (adjusted *p* = 0.0031), and 96 h (adjusted *p* < 0.0001), with no significant difference at 24 h (adjusted *p* = 0.9947). These findings indicate cell- and context-dependent effects of SPINT2 modulation on CCK-8 readouts.

### 3.13. Effect of SPINT2 on Wound Closure in Liver Cancer Cells

A scratch assay was used to evaluate the effect of SPINT2 on wound closure. Data showed that the 97H OE overexpression group exhibited obvious healing trends at both 24 h and 48 h. Compared with the control group, wound closure was significantly increased at 24 h (*p* = 0.0055) and 48 h (*p* < 0.0001). In the 97H SH group, wound closure was significantly reduced compared with the corresponding control at both 24 h (*p* = 0.0185) and 48 h (*p* = 0.0374) (Figure 4C). In LM3 cells, no significant difference was observed at 24 h for either OE (*p* = 0.6824) or SH (*p* = 0.2508), whereas at 48 h wound closure was significantly increased in the OE group (*p* = 0.0348) and reduced in the SH group (*p* = 0.0033) compared with the corresponding controls. Because cell proliferation was not independently controlled in this assay, the wound-healing results cannot be attributed exclusively to cell migration.

### 3.14. Effect of SPINT2 on the Sphere-Forming Ability of Liver Cancer Cells

After culturing the MHCC97H cell line for 6 days and the LM3 liver cancer cell line for 7 days, tumor spheres were photographed and counted for the MHCC97H and LM3 liver cancer cell lines. It was found that the number of tumor spheres in the OE group cells of the 97H and LM3 cell lines was significantly greater than that in the corresponding control NC group, with statistically significant differences (*p* < 0.05, Figure 5), indicating that SPINT2 overexpression significantly increased sphere-forming capacity in MHCC97H and LM3 cells. The number of tumor spheres in the SH group cells of the 97H and LM3 cell lines was significantly less than that in the corresponding control NC group, with statistically significant differences (*p* < 0.05, Figure 5B), indicating that SPINT2 knockdown significantly reduced sphere-forming capacity in MHCC97H and LM3 cells. This also suggests that the upregulated expression of the SPINT2 gene in DPHCC tumor tissues may be associated with increased sphere-forming capacity.

### 3.15. Effect of SPINT2 on Xenograft Tumor Growth

To evaluate the effect of SPINT2 on xenograft tumor growth, this study employed a subcutaneous xenograft model in nude mice. First, 97H OE (SPINT2 overexpression), 97H SH (SPINT2 knockdown), LM3 OE (SPINT2 overexpression), and LM3 SH (SPINT2 knockdown) cell lines were constructed and compared with their respective control cell lines. Subsequently, these cell lines were injected subcutaneously into nude mice to observe tumor formation conditions and growth rates.

Experimental results showed that xenograft tumor growth in the 97H OE group was significantly increased, and there was a significant difference in tumor volume compared to the control group (*p* < 0.05). This result indicates that SPINT2 overexpression may promote the proliferation and growth of tumor cells, suggesting its potential promoting role in tumor formation. Conversely, xenograft tumor growth in the 97H SH group was significantly reduced, and the tumor growth rate was significantly weakened compared to the control group (*p* < 0.05), which further supports an effect of SPINT2 knockdown on short-term xenograft growth in MHCC-97H cells. For the LM3 cell line, LM3 OE and LM3 SH did not show significant differences in xenograft tumor growth compared to their respective control cell lines (*p* > 0.05), as shown in Figure 5. The tumor formation situation of each cell line after sacrificing the nude mice is shown in Figure 5A. This result may indicate that the effect of SPINT2 on tumor formation exists heterogeneity in different cellular contexts.

## 4. Discussion

HCC is a leading cause of cancer-related mortality worldwide, characterized by significant intra-tumoral heterogeneity that contributes to treatment resistance and poor clinical outcomes. This heterogeneity is partly driven by the presence of cancer stem cells (CSCs), a subpopulation of tumor cells with self-renewal and differentiation capabilities that are strongly implicated in tumor initiation, progression, and recurrence [11]. Within the spectrum of primary liver cancers, combined hepatocellular–cholangiocarcinoma (cHCC-CCA), which exhibits features of both hepatocytic and cholangiocytic differentiation, represents a particularly aggressive entity with unfavorable tumor biology. However, the prevalence of DPHCC cells within otherwise conventional HCC, their clinical significance, and the molecular correlates associated with this aggressive phenotype remain poorly understood [17,18,19], representing a critical gap in our knowledge that hinders the development of effective prognostic biomarkers and targeted therapies.

In this study, we characterized a dual-lineage HCC phenotype in a cohort of 83 patients using multiplex immunofluorescence, complemented by single-cell RNA sequencing and CyTOF profiling. Using the primary >5% operational cutoff, 19 of 83 tumors (22.9%) were classified as DPHCC-positive. These cases showed adverse clinicopathological and survival features in the primary cohort. Single-cell analyses further identified DPHCC-associated transcriptional and phenotypic states enriched for stemness- and EMT-related features, while SPINT2 emerged as a candidate gene associated with these states. Functional perturbation experiments showed context-dependent effects of SPINT2 modulation on malignant tumor-cell phenotypes. Taken together, these findings support DPHCC as a clinically and biologically relevant dual-lineage phenotypic state within HCC rather than establishing it as a completely discrete molecular subtype.

The DPHCC phenotype identified in the present study is related to, but not synonymous with, CK19-positive or progenitor-like HCC. CK19 positivity is associated with biliary/progenitor differentiation in HCC [20]; however, CK19 expression alone does not demonstrate simultaneous hepatocytic and cholangiocytic differentiation within the same tumor cell. Previous work explicitly separated DPHCC from CK19-positive HCC by requiring same-cell co-expression of hepatocytic and cholangiocytic markers [14,16]. That study also distinguished DPHCC from tumors containing spatially separate hepatocellular and cholangiocarcinoma components or non-overlapping lineage-marker expression [16]. We therefore do not regard DPHCC as a completely separate lineage-defined subtype; rather, it represents a dual-lineage immunophenotypic state that may partially overlap with CK19-positive/progenitor-like HCC but is distinguished by concurrent hepatocytic and cholangiocytic features within the same tumor cells. Whether DPHCC represents a stable tumor subtype or a transient phenotypic state remains unresolved. Given its partial overlap with progenitor-like programs and the observed enrichment of stemness- and EMT-related features, our findings are compatible with a role for tumor-cell plasticity, but the present cross-sectional data cannot determine whether individual tumor cells dynamically transition into or out of the DPHCC state. Previous DPHCC studies have used more stringent quantitative criteria, including a >15% dual-positive-cell threshold [14,16]; therefore, the >5% threshold used in the present study should be regarded as a study-specific operational cutoff rather than a universal pathological standard. Our finding that DPHCC cells are enriched in stemness-related pathways, including the “Desert Stem Cell HCC Subclass Up” and EMT pathways [21], supports an association between this cellular state and stemness-related biology. CSCs are known to drive tumor initiation, recurrence, and resistance to therapy [22,23,24], and our data suggest that the DPHCC phenotype shows CSC-like molecular features within HCC [25]. The enrichment of EMT pathways is consistent with the aggressive behavior and poor clinical outcomes observed in DPHCC patients, as EMT is a key process enabling invasion and metastasis [26,27]. These results position DPHCC not merely as a histological curiosity but as a biologically relevant state that is associated with aggressive tumor behavior [28,29], providing biological context for its poor prognosis and suggesting that CSC- and EMT-related pathways may warrant further investigation in this patient subset [26].

Functional evaluation of SPINT2 revealed complex and context-dependent effects of SPINT2 modulation on tumor-cell behavior. Our in vitro data demonstrate that SPINT2 modulation affects invasion, proliferation-related phenotypes, wound closure, and sphere-forming capacity, with context-dependent effects across assays and cell lines. These context-dependent phenotypic effects are notable given that SPINT2 is traditionally viewed as a tumor suppressor in many contexts [30,31,32], where it inhibits the HGF/MET signaling pathway [33,34]. The observed co-expression of SPINT2 with CK19 raises the hypothesis that SPINT2 may be related to cellular plasticity and lineage-associated programs; however, the present experiments do not establish that SPINT2 induces, maintains, or reverses the dual-lineage phenotype. Accordingly, the present functional assays should be interpreted as evaluating malignant tumor-cell phenotypes rather than directly testing regulation of DPHCC identity. The differential responses of MHCC-97H and MHCC-LM3 cells to SPINT2 modulation further suggest that the biological effects of SPINT2 are strongly dependent on cellular context. Importantly, MHCC-LM3 was established through repeated in vivo selection of pulmonary metastatic lesions derived from MHCC97-H, indicating that these two cell models share a related ancestral background but represent distinct metastasis-selected states rather than completely unrelated genetic backgrounds [35]. Previous studies using the MHCC97-L–MHCC97-H–HCC-LM3 stepwise metastatic model have demonstrated progressive molecular alterations associated with metastatic potential, including changes in the miR-192/SLC39A6/SNAIL axis, which regulates epithelial–mesenchymal transition and invasive behavior [36]. These pre-existing differences in metastatic and EMT-related signaling may therefore influence the dependence of each cell line on SPINT2-regulated pathways. SPINT2 is known to modulate protease-dependent HGF/MET signaling, while activated MET can engage downstream pathways including PI3K/AKT, RAS/MAPK, RAC1, and other motility-associated signaling networks [34,37]. Consequently, differences in baseline pathway activity, pathway dependency, or compensatory signaling between MHCC-97H and MHCC-LM3 may contribute to their differential sensitivity to SPINT2 overexpression or knockdown. However, these signaling pathways were not directly compared between the two cell lines in the present study; therefore, this interpretation should be regarded as a biologically plausible explanation rather than a demonstrated mechanism, and further mechanistic studies will be required to clarify the basis of this context-dependent response. Overall, these results support an association between SPINT2 and aggressive tumor-cell phenotypes, including sphere-forming capacity, but do not establish SPINT2 as a direct driver of the DPHCC state.

While our study does not directly address immune mechanisms, the observed association of DPHCC with a poor prognosis [28] and its association with stemness- and EMT-related pathways may have potential implications for the tumor microenvironment and anti-tumor immunity [38,39,40]. CSCs, including those with a DPHCC phenotype, are known to actively shape an immunosuppressive microenvironment [41,42,43] by secreting cytokines and chemokines that recruit regulatory T cells (Tregs) and myeloid-derived suppressor cells (MDSCs), while also downregulating antigen presentation machinery. These potential immune implications are speculative and were not directly tested in the present study. The prognostic value of the platelet-to-lymphocyte ratio (PLR) in our cohort, identified as an independent risk factor, is also consistent with a possible relationship between systemic inflammation and adverse outcome, as PLR is a systemic marker of inflammation and immune dysfunction that has been linked to poor outcomes in HCC. However, the present study did not directly evaluate treatment response, and our findings do not establish DPHCC as a predictive biomarker for immunotherapy or other treatment selection. Prior retrospective evidence has suggested a potential benefit of adjuvant TACE after resection in patients with DPHCC [13], but prospective validation is required before DPHCC status can be used to guide treatment decisions.

Several limitations warrant acknowledgment in interpreting our findings. First, the relatively small sample size of the clinical cohort (*n* = 83) and the single-center design may introduce selection bias and limit the generalizability of our conclusions regarding DPHCC prevalence and prognosis [16]; validation in larger, multi-institutional cohorts is imperative. SPINT2 immunohistochemical assessment was performed in the same 83-patient cohort and therefore does not constitute independent external validation. The CyTOF analysis was also limited to a 16-patient subset and should therefore be considered exploratory. Second, although SPINT2 modulation affected several aggressive tumor-cell phenotypes, the precise downstream signaling cascades mediating these effects remain unclear, and rescue experiments were not performed in the present study; further mechanistic studies will therefore be required. In addition, the downstream functional phenotypes were evaluated using a single selected shRNA construct rather than multiple independent shRNAs; therefore, potential off-target effects cannot be completely excluded. The baseline full dual-lineage marker profile of MHCC-97H and MHCC-LM3 cells and changes in these markers following SPINT2 perturbation were not evaluated. Third, potential batch effects arising from the integration of diverse high-dimensional datasets (scRNA-seq, CyTOF, multiplex IF) could influence clustering outcomes, despite rigorous computational correction. Finally, the functional heterogeneity observed between cell lines in vivo underscores the context-dependency of SPINT2, suggesting that its phenotypic effects may be modulated by specific genetic backgrounds or microenvironmental cues not fully captured in our current models [31,32]. Moreover, the xenograft experiments included only four mice per group and used a short 15-day observation period; these findings should therefore be considered exploratory and interpreted as short-term xenograft growth rather than definitive evidence of long-term tumorigenic capacity.

## 5. Conclusions

In conclusion, this study characterizes DPHCC as a clinically relevant dual-lineage phenotypic state characterized by co-expression of hepatocytic and cholangiocytic markers and enriched stemness-related features. SPINT2 emerged as a candidate molecule associated with the DPHCC-related state, and functional perturbation experiments showed context-dependent effects on malignant tumor-cell phenotypes, including invasion, proliferation, sphere-forming capacity, and tumor growth. The association of SPINT2 expression with DPHCC and adverse survival supports its potential biological and prognostic relevance, but the present data do not establish SPINT2 as a validated independent biomarker or as a direct molecular driver of the dual-lineage phenotype. Further mechanistic studies and validation in larger, independent cohorts are required to determine whether SPINT2 has clinical utility as a prognostic marker or therapeutic target in DPHCC.

## Figures and Tables

**Figure 1:**
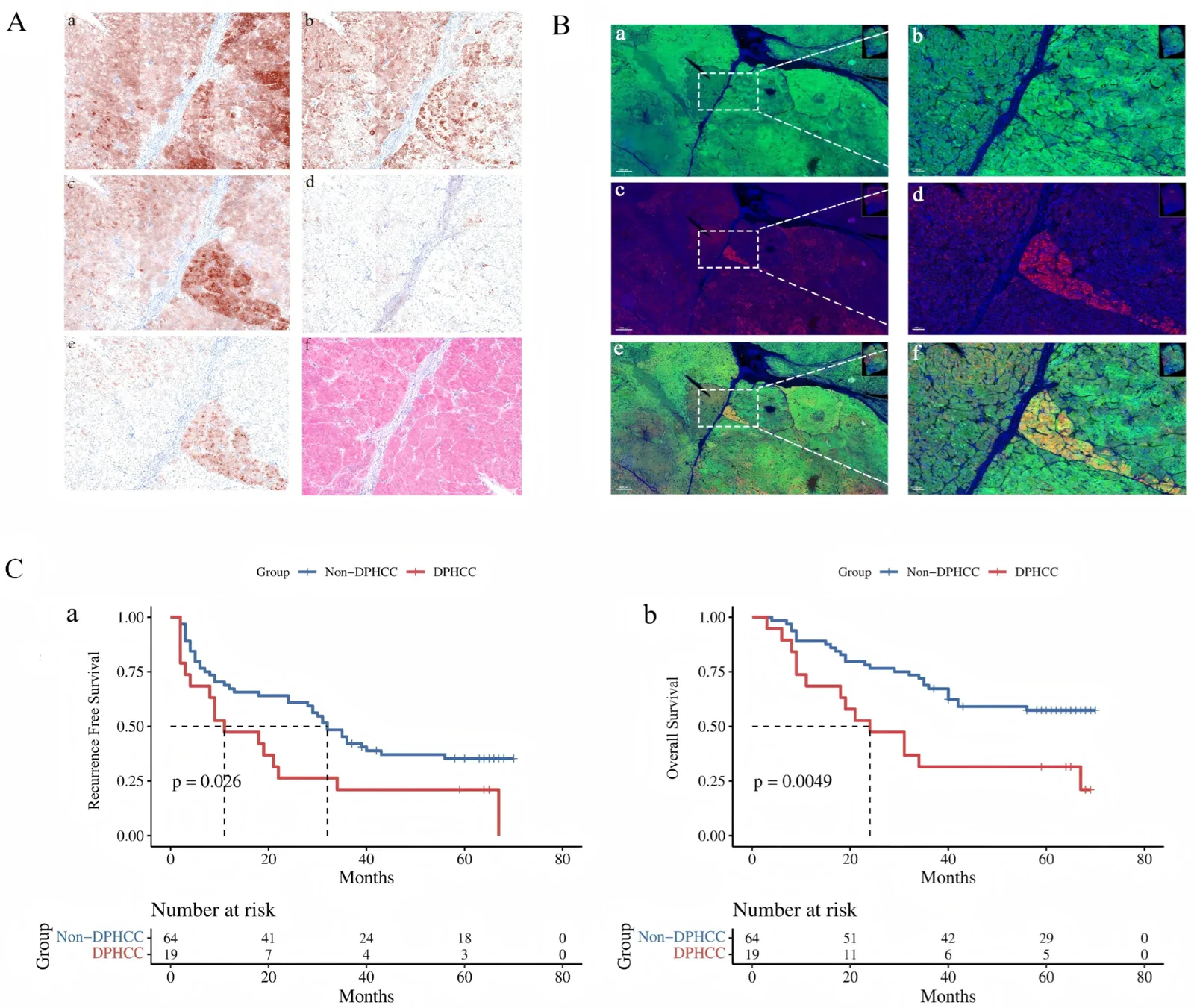
Identification of dual-phenotype HCC (DPHCC) and patient prognosis. (**A**) Multiplex immunofluorescence staining simulating immunohistochemical chromogenic detection and H&E staining (×200). The panels show staining for: (**a**) Hep Par1, (**b**) Arginase-1, (**c**) Glypican-3, (**d**) CK7, (**e**) CK19, and (**f**) H&E. (**B**) Visualization of DPHCC cells: green fluorescence indicates positivity for hepatocyte markers ((**a**): Hep Par1, Arginase-1, Glypican-3); red fluorescence indicates positivity for cholangiocyte markers ((**c**): CK7, CK19). DPHCC cells co-expressing both markers appear yellow in merged images. Panel (**e**) represents the merged image of red and green fluorescent signals. Scale bars represent 600 μm (**a**,**c**,**e**) in the low-magnification fluorescence images and 100 μm (**b**,**d**,**f**) in the corresponding enlarged views. (**C**) Survival analysis comparing DPHCC and non-DPHCC patients: (**a**) recurrence-free survival (RFS) curves; (**b**) overall survival (OS) curves. Survival differences in panel C were assessed using the log-rank test. DPHCC, dual-phenotype hepatocellular carcinoma; RFS, recurrence-free survival; OS, overall survival.

**Figure 2:**
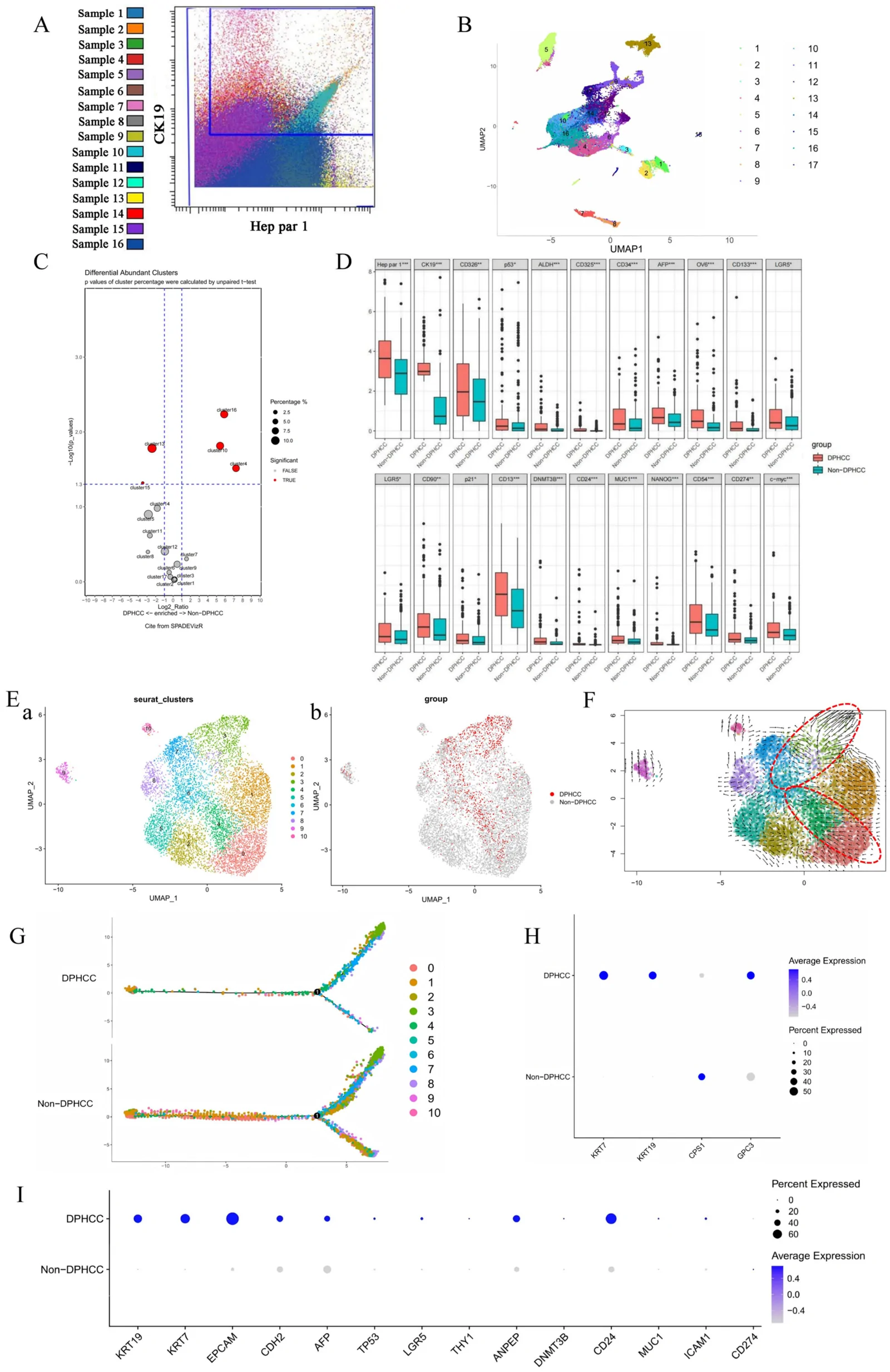
Single-cell transcriptomic and mass cytometry (CyTOF) profiling of DPHCC. (**A**) CyTOF analysis of tumor cells from 16 samples. The plot displays Hep Par1 expression (x-axis) versus CK19 expression (y-axis). DPHCC tumor cells, defined by dual positivity for Hep Par1 and CK19, are enclosed within the blue rectangle. (**B**) UMAP visualization of all tumor cells from CyTOF data, clustered into 17 distinct groups based on cancer stem cell (CSC) marker expression profiles. (**C**) Volcano plot illustrating the correlation between the 17 clusters and DPHCC tumor cells. DPHCC cells are predominantly enriched in Clusters 13 and 15. (**D**) Statistically significant differences in CSC marker expression between DPHCC and non-DPHCC tumor cells across clusters. * *p* < 0.05, ** *p* < 0.01, *** *p* < 0.001. (**E**) Single-cell RNA sequencing (scRNA-seq) analysis of tumor cells from 8 HCC tissues: (**a**) UMAP plot showing 11 clusters based on CSC expression signatures; (**b**) the broader DPHCC-associated transcriptional state (highlighted in red) identified based on hepatocytic and cholangiocytic lineage-marker expression. (**F**) Velocyto RNA velocity vector plot. Arrow direction represents the inferred transcriptional direction, and vector magnitude reflects the estimated RNA-velocity magnitude. DPHCC-enriched clusters are outlined with a red dashed circle. (**G**) Monocle pseudotime analysis of DPHCC versus non-DPHCC tumor cells. (**H**) Differential expression of hepatocyte and cholangiocyte markers used for DPHCC identification between DPHCC and non-DPHCC tumor cells. (**I**) Transcriptomic-level differences in CSC marker expression between DPHCC and non-DPHCC tumor cells. CyTOF analysis included 16 patient-derived tumor samples, and scRNA-seq analysis included eight patient-derived tumor samples. DPHCC, dual-phenotype hepatocellular carcinoma; CyTOF, cytometry by time of flight; scRNA-seq, single-cell RNA sequencing.

**Figure 3:**
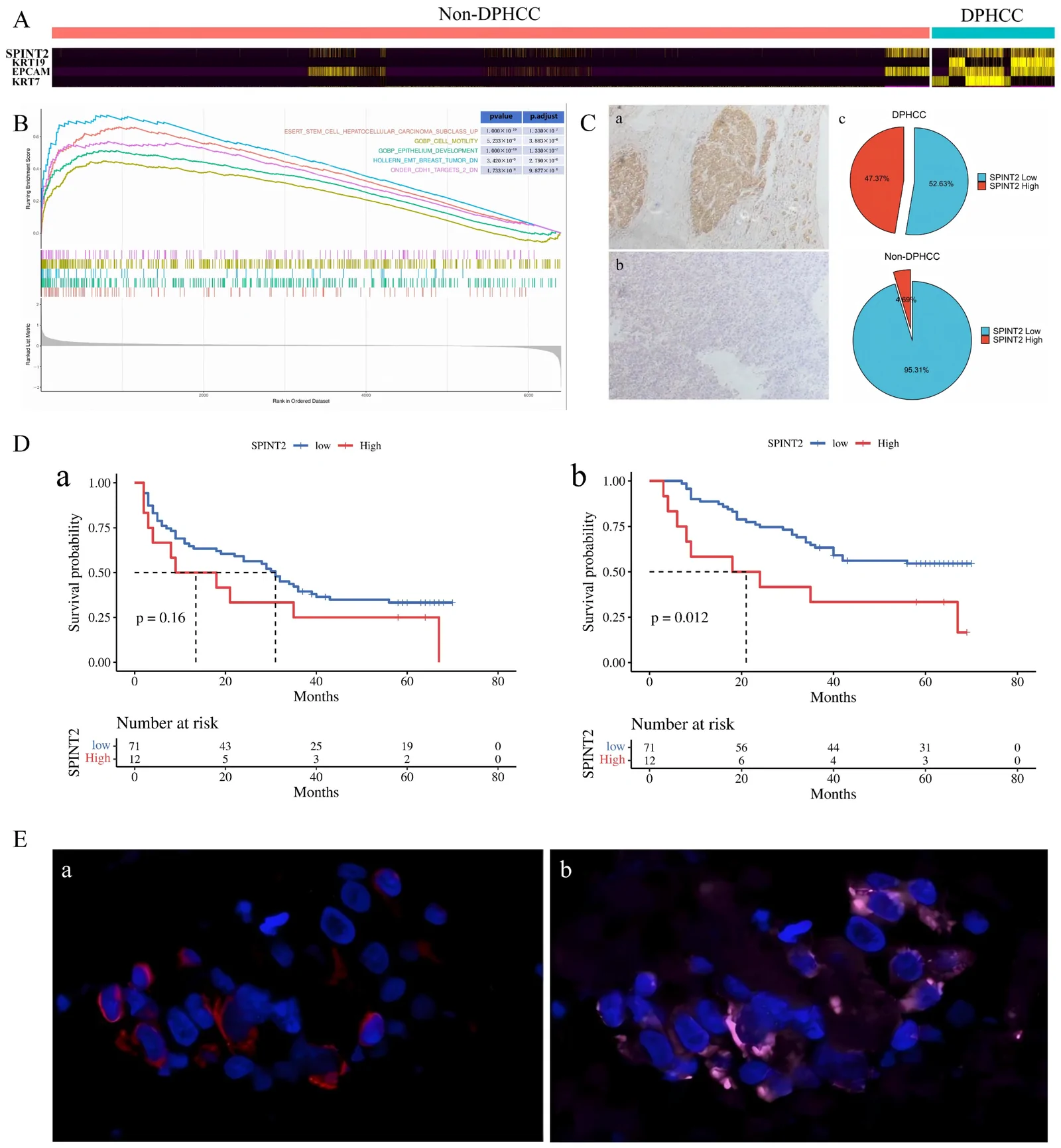
SPINT2 is closely associated with DPHCC. (**A**) Heatmap of the top 4 differentially expressed genes between DPHCC and non-DPHCC tumor cells, with SPINT2 identified among the top candidates in the original cell-level analysis. (**B**) Gene Set Enrichment Analysis (GSEA) of pathways enriched in DPHCC tumor cells, highlighting pathways enriched in the DPHCC-associated state. (**C**) SPINT2 protein expression in HCC tissues: (**a**) representative high expression; (**b**) representative low expression; (**c**) proportion of SPINT2-high cases in DPHCC versus non-DPHCC tumor tissues. SPINT2 expression was evaluated in 83 HCC tissues. (**D**) According to the expression level of SPINT2, patients were divided into SPINT2 high-expression and low-expression groups, and survival analysis was performed to compare RFS (**a**) and OS (**b**) between the two groups. Survival differences were assessed using the log-rank test. (**E**) Dual immunofluorescence staining showing co-expression of CK19 ((**a**) red) and SPINT2 ((**b**) pink) in tumor cells, blue indicates nuclear DAPI staining. RFS, recurrence-free survival; OS, overall survival.

**Figure 4:**
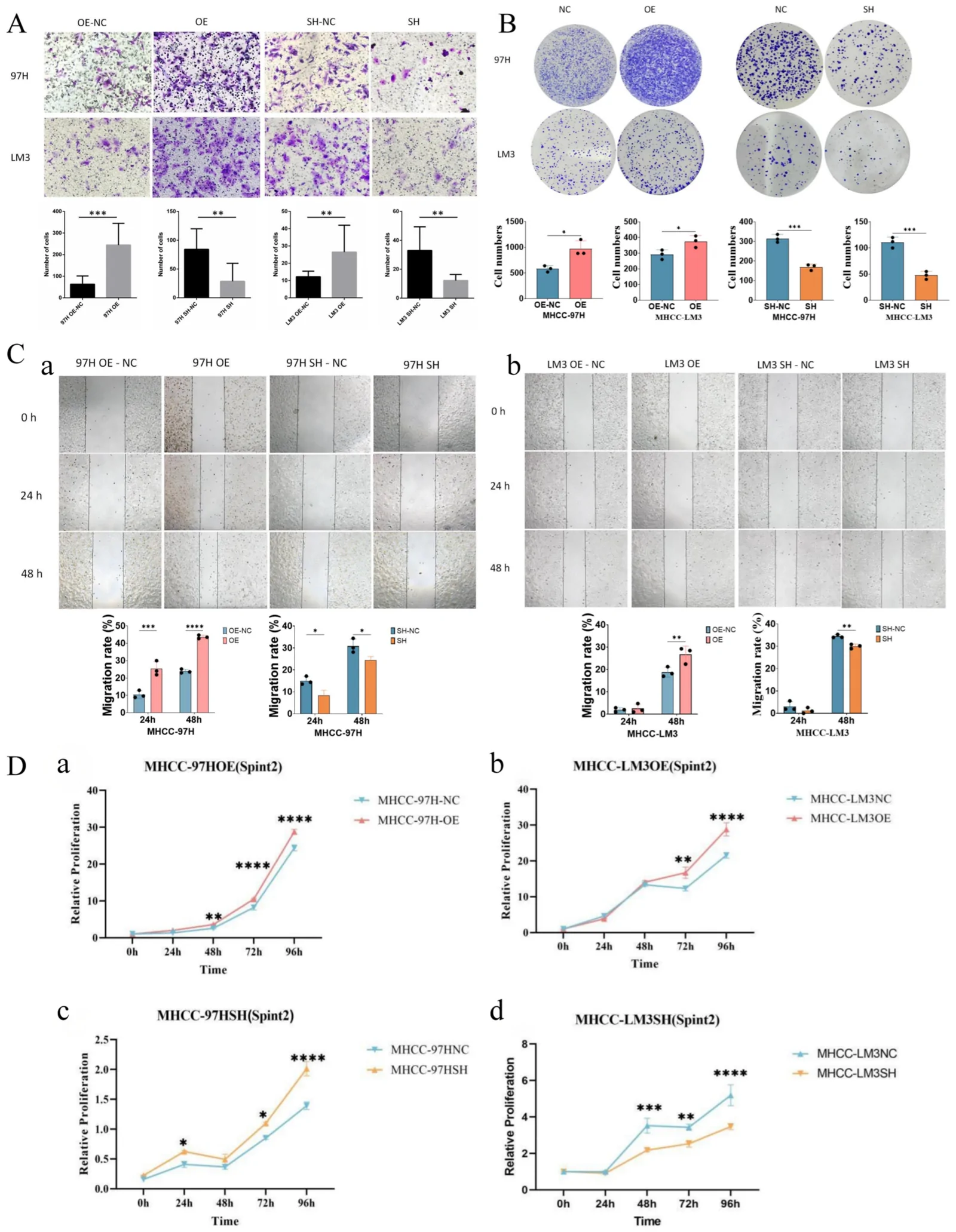
In vitro functional validation of SPINT2 in HCC cells. (**A**) Transwell invasion assay showing changes in invasive ability after SPINT2 overexpression or knockdown in MHCC-97H and MHCC-LM3 cell lines. (**B**) Colony formation assay evaluating proliferative capacity after SPINT2 modulation in MHCC-97H and MHCC-LM3 cells. (**C**) Wound healing (scratch) assay assessing wound closure following SPINT2 overexpression or knockdown. Panels (**a**,**b**) show the scratch assay results of MHCC-97H and MHCC-LM3 cells, respectively. (**D**) CCK-8 proliferation assay measuring cell viability after SPINT2 manipulation in MHCC-97H and MHCC-LM3 cells. OE indicates SPINT2 overexpression (**a**,**b**), SH indicates SPINT2 knockdown (**c**,**d**), and NC indicates the corresponding negative control. Transwell invasion was assessed after 24 h, colony formation after 16 days, wound closure at 0, 24, and 48 h, and CCK-8 measurements at 0, 24, 48, 72, and 96 h. Data are presented as mean ± SD from three independent biological replicates. Transwell invasion, colony-formation, and wound-healing comparisons were assessed using two-tailed unpaired Student’s *t*-tests, whereas CCK-8 time-course data were analyzed using two-way ANOVA followed by Šídák’s multiple-comparisons test. * *p* < 0.05, ** *p* < 0.01, *** *p* < 0.001, and **** *p* < 0.0001.

**Figure 5:**
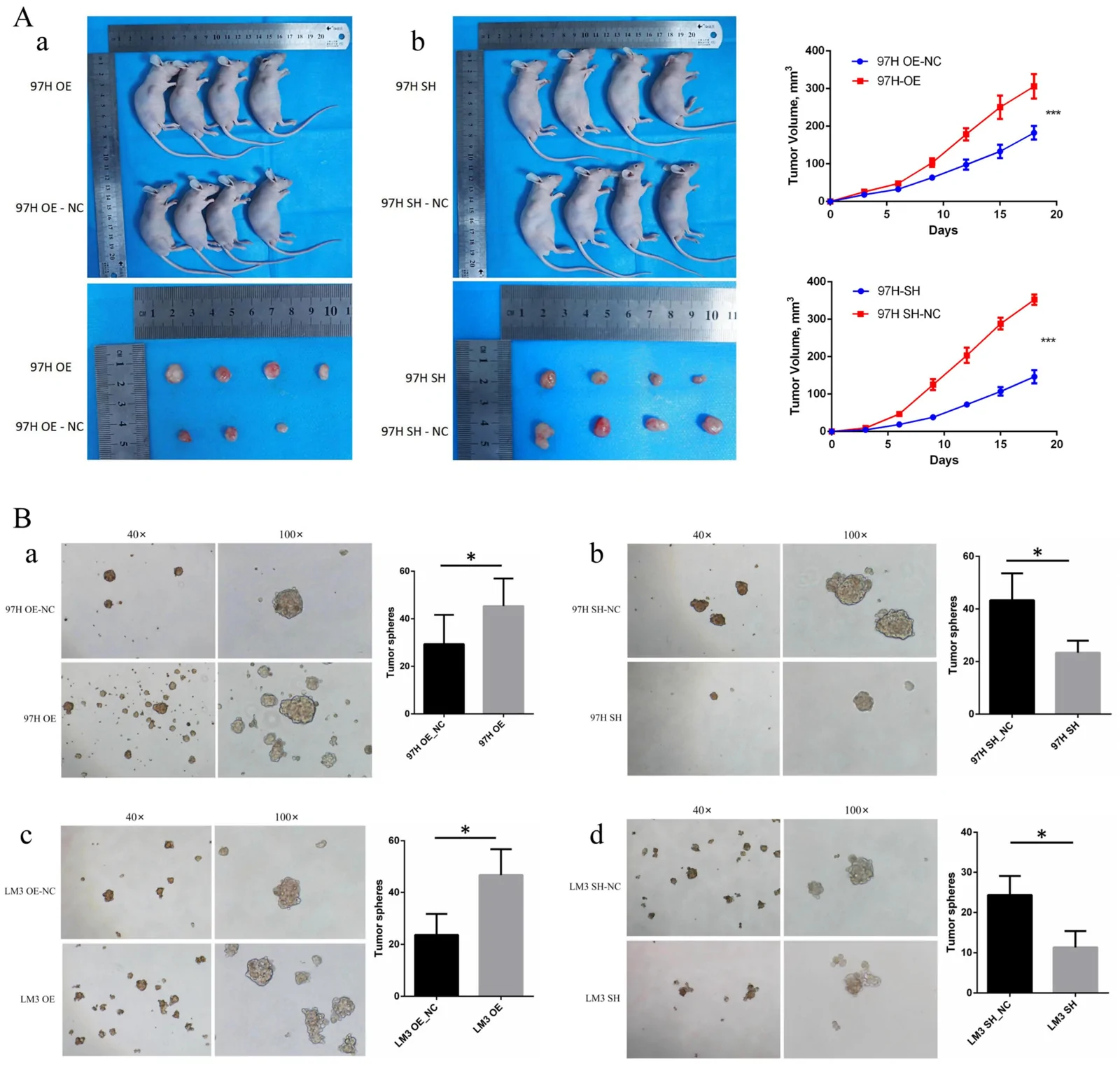
Effects of SPINT2 modulation on in vivo tumor growth and sphere-forming capacity. (**A**) Subcutaneous xenograft tumor formation in nude mice: tumor growth curves and representative images after SPINT2 overexpression or knockdown in HCC cell lines. Panels (**a**,**b**) show subcutaneous xenograft tumor formation in nude mice using MHCC-97H cells with SPINT2 overexpression and knockdown, respectively. (**B**) Tumor sphere formation assay assessing sphere-forming capacity after SPINT2 modulation in HCC cell lines. Panels (**a**–**d**) show the results of MHCC-97H OE, MHCC-97H SH, MHCC-LM3 OE, and MHCC-LM3 SH cells, respectively. For xenograft experiments, *n* = 4 mice were included per group. Tumor volume was measured every 3–4 days and calculated as (length × width^2^)/2; plotted values represent mean tumor volume ± SD. All four animals and the corresponding excised tumors in each group are shown in the endpoint images. For sphere-forming assays, spheres > 50 μm were counted after 6–7 days in three independent biological replicates. Representative sphere images are shown at 40× and 100× magnification. Data are presented as mean ± SD. Statistical comparisons between two groups were performed using two-tailed unpaired Student’s *t*-tests. * *p* < 0.05 and *** *p* < 0.001.

**Table 1:** Baseline characteristics of 83 HCC patients stratified by DPHCC and non-DPHCC groups (*N* = 83).

Variables	DPHCC	Non-DPHCC	*p*-Value
	(*N* = 19)	(*N* = 64)	
Male	17	57	0.999
Age ≥ 60 years	2	24	0.026
BMI ≥ 24	9	21	0.246
Hypertension	3	10	0.999
Diabetes mellitus	0	5	0.479
Incomplete tumor capsule	3	3	0.256
Multiple nodules	6	30	0.237
Maximum tumor diameter > 5 cm	12	44	0.648
Macrovascular invasion	6	9	0.161
BCLC stage, A/B/C	11/2/6	33/22/9	0.065
Clonorchiasis sinensis	0	9	0.083
Microvascular invasion (MVI)	12	35	0.513
Edmondson grade, III–IV	11	28	0.278
Liver cirrhosis	8	26	0.908
HBsAg positive	17	51	0.330
NLR ≥ 2.51	5	15	0.797
PLR ≥ 180	3	13	0.914
AFP ≥ 400 ng/mL	12	20	0.012
PT > 13.0 s	6	11	0.298
TBIL > 21 μmol/L	3	13	0.914
Albumin < 35 g/L	4	16	0.962
ALT > 40 U/L	10	35	0.874
AST > 40 U/L	10	40	0.440

**Table 2:** Univariate and multivariate analysis of factors affecting recurrence in 83 HCC patients.

Variables	Univariate Analysis	*p*-Value	Multivariate Analysis	*p*-Value
	HR (95% CI)		HR (95% CI)	
Male	1.106 (0.474–2.580)	0.816		
Age ≥ 60 years	0.727 (0.407–1.296)	0.279		
BMI ≥ 24	0.678 (0.390–1.178)	0.168		
Hypertension	1.883 (0.807–4.390)	0.143		
Diabetes mellitus	0.987 (0.356–2.737)	0.981		
Incomplete tumor capsule	1.889 (0.748–4.769)	0.178		
Multiple nodules	2.855 (1.662–4.907)	<0.001	4.325 (2.335–8.009)	<0.001
Tumor diameter ≥ 5 cm	1.087 (0.626–1.887)	0.766		
Macrovascular invasion	1.475 (0.740–2.940)	0.270		
Microvascular invasion	1.580 (0.899–2.777)	0.111		
Edmondson grade, III–IV	1.751 (1.037–2.957)	0.036	0.973 (0.545–1.735)	0.925
Liver cirrhosis	1.095 (0.649–1.849)	0.734		
HBsAg positive	1.481 (0.782–2.809)	0.228		
NLR ≥ 2.51	1.726 (1.008–2.954)	0.047	1.054 (0.567–1.960)	0.869
PLR ≥ 180	2.998 (1.615–5.563)	0.001	4.976 (2.228–11.112)	0.001
AFP ≥ 400 ng/mL	1.286 (0.746–2.219)	0.365		
PT > 13.0 s	1.627 (0.873–3.033)	0.125		
TBIL > 21 μmol/L	1.450 (0.777–2.706)	0.242		
Albumin > 35 g/L	0.872 (0.488–1.557)	0.643		
ALT > 40 U/L	1.499 (0.891–2.519)	0.127		
AST > 40 U/L	1.421 (0.818–2.468)	0.212		
DPHCC phenotype	1.893 (1.058–3.389)	0.032	3.218 (1.643–6.303)	0.001

**Table 3:** Univariate and multivariate analysis of factors affecting overall survival (OS) in 83 HCC patients.

Variables	Univariate Analysis	*p*-Value	Multivariate Analysis	*p*-Value
	HR (95% CI)		HR (95% CI)	
Male	1.697 (0.712–4.046)	0.233		
Age ≥ 60 years	0.917 (0.475–1.771)	0.796		
BMI ≥ 24	0.650 (0.336–1.256)	0.200		
Hypertension	1.423 (0.558–3.627)	0.460		
Diabetes mellitus	1.320 (0.318–5.475)	0.702		
Incomplete tumor capsule	2.969 (1.147–7.681)	0.025	2.756 (0.998–7.616)	0.051
Multiple nodules	1.766 (0.955–3.266)	0.07		
Tumor diameter ≥ 5 cm	0.954 (0.494–1.842)	0.888		
Macrovascular invasion	1.453 (0.669–3.157)	0.345		
Microvascular invasion	1.590 (0.847–2.983)	0.149		
Edmondson grade, III–IV	3.104 (1.617–5.958)	0.001	2.28 (1.138–4.568)	0.02
Liver cirrhosis	1.234 (0.668–2.282)	0.502		
HBsAg positive	0.986 (0.436–2.231)	0.974		
NLR ≥ 2.51	1.190 (0.623–2.272)	0.598		
PLR ≥ 180	2.931 (1.484–5.791)	0.002	2.974 (1.427–6.199)	0.004
AFP ≥ 400 ng/mL	1.977 (1.063–3.674)	0.031	1.333 (0.692–2.567)	0.39
PT > 13.0 s	1.687 (0.843–3.375)	0.139		
TBIL > 21 μmol/L	1.894 (0.947–3.789)	0.071		
Albumin > 35 g/L	0.668 (0.345–1.294)	0.232		
ALT > 40 U/L	1.299 (0.704–2.398)	0.402		
AST > 40 U/L	1.672 (0.864–3.237)	0.127		
DPHCC phenotype	2.448 (1.277–4.693)	0.007	2.153 (1.069–4.336)	0.032

## Data Availability

The processed data supporting the findings of this study are provided within the article and Supplementary Materials. Additional de-identified data may be made available by the corresponding author upon reasonable request and in accordance with institutional and ethical requirements.

## Supplementary Materials

The following supporting information can be downloaded at: https://www.mdpi.com/article/10.3390/cancers18193102/s1, Table S1: Patient-specific contributions to the DPHCC-associated and non-DPHCC transcriptional states in the scRNA-seq dataset; Table S2: Donor-aware pseudobulk analysis of SPINT2 expression in the DPHCC-associated transcriptional state.

## Abbreviations

The following abbreviations are used in this manuscript:

HCC	Hepatocellular carcinoma
cHCC-CCA	Combined Hepatocellular–Cholangiocarcinoma
DPHCC	Dual-phenotype HCC
SPINT2	Serine protease inhibitor Kunitz type 2
scRNA-seq	Single-cell RNA sequencing
RFS	Recurrence-free survival
OS	Overall survival
mIF	Multiplex Immunofluorescence
CyTOF	Mass Cytometry
IHC	Immunohistochemistry
Hep1	Hep par 1
GPC-3	Glypican-3
Arg-1	Arginase-1
