# Supplementary material for: SPINT2 Is Associated with Dual-Phenotype Hepatocellular Carcinoma and Aggressive Tumor Features
Source: Cancers (Basel). 2026 Sep 24;18(19):3102. doi: 10.3390/cancers18193102 (PMC13664038; doi:10.3390/cancers18193102)
Supplement: Supplementary file 1 [file cancers-18-03102-s001.zip › cancers-4494728-supplementary.pdf]

Supplementary Table S1. Patient-specific contributions to the DPHCC-associated and non-DPHCC transcriptional states in the scRNA-seq dataset.

Patient	Total malignant cells	DPHCC-associated cells	non-DPHCC cells	DPHCC-associated fraction (%)
CA185009	2,421	150	2,271	6.2
CA185195	579	145	434	25.0
CA186580	212	2	210	0.9
CA187579	394	344	50	87.3
CA188236	880	15	865	1.7
CA188998	983	22	961	2.2
CA189155	2,618	20	2,598	0.8
CA189470	786	391	395	49.7
**Total**	**8,873**	**1,089**	**7,784**	**12.3**

Abbreviations: DPHCC, dual-phenotype hepatocellular carcinoma; scRNA-seq, single-cell RNA sequencing. The DPHCC-associated state refers to the broader transcriptional state used in the original single-cell analysis and should not be interpreted as indicating that every cell fulfilled the stricter same-cell dual-lineage criterion.

Supplementary Table S2. Donor-aware pseudobulk analysis of SPINT2 expression in the DPHCC-associated transcriptional state.

Analysis	Patients included	log2FC	logCPM	F statistic	P value	FDR
Primary donor-aware pseudobulk analysis	8	0.599	6.402	4.723	0.0298	0.630
Sensitivity analysis requiring ≥20 cells in each state per patient	6	0.621	6.609	7.577	0.0076	0.561

Abbreviations: DPHCC, dual-phenotype hepatocellular carcinoma; log2FC, log2 fold change; logCPM, log counts per million; FDR, false discovery rate. Raw counts were aggregated by patient and transcriptional state, and differential expression was evaluated using edgeR with patient identity included in the design matrix. Positive log2FC values indicate higher SPINT2 expression in the DPHCC-associated state. Although the direction of association was consistent across analyses, SPINT2 did not reach transcriptome-wide significance after FDR correction.
